# Highly Overexpressed AtC3H18 Impairs Microgametogenesis *via* Promoting the Continuous Assembly of mRNP Granules

**DOI:** 10.3389/fpls.2022.932793

**Published:** 2022-07-15

**Authors:** Liai Xu, Tingting Liu, Xingpeng Xiong, Xiuping Shen, Li Huang, Youjian Yu, Jiashu Cao

**Affiliations:** ^1^Laboratory of Cell & Molecular Biology, Institute of Vegetable Science, Zhejiang University, Hangzhou, China; ^2^Collaborative Innovation Center for Efficient and Green Production of Agriculture in Mountainous Areas of Zhejiang Province, College of Horticulture Science, Zhejiang A&F University, Hangzhou, China

**Keywords:** AtC3H18, pollen development, mRNP granules, processing bodies (PBs), stress granules (SGs), *Arabidopsis thaliana*

## Abstract

Plant CCCH zinc-finger proteins form a large family of regulatory proteins function in many aspects of plant growth, development and environmental responses. Despite increasing reports indicate that many CCCH zinc-finger proteins exhibit similar subcellular localization of being localized in cytoplasmic foci, the underlying molecular mechanism and the connection between this specific localization pattern and protein functions remain largely elusive. Here, we identified another cytoplasmic foci-localized CCCH zinc-finger protein, AtC3H18, in *Arabidopsis thaliana*. *AtC3H18* is predominantly expressed in developing pollen during microgametogenesis. Although *atc3h18* mutants did not show any abnormal phenotype, possibly due to redundant gene(s), aberrant *AtC3H18* expression levels caused by overexpression resulted in the assembly of AtC3H18-positive granules in a dose-dependent manner, which in turn led to male sterility phenotype, highlighting the importance of fine-tuned *AtC3H18* expression. Further analyzes demonstrated that AtC3H18-positive granules are messenger ribonucleoprotein (mRNP) granules, since they can exhibit liquid-like physical properties, and are associated with another two mRNP granules known as processing bodies (PBs) and stress granules (SGs), reservoirs of translationally inhibited mRNAs. Moreover, the assembly of AtC3H18-positive granules depends on mRNA availability. Combined with our previous findings on the *AtC3H18* homologous genes in *Brassica campestris*, we concluded that appropriate expression level of *AtC3H18* during microgametogenesis is essential for normal pollen development, and we also speculated that AtC3H18 may act as a key component of mRNP granules to modulate pollen mRNAs by regulating the assembly/disassembly of mRNP granules, thereby affecting pollen development.

## Introduction

Modulation of gene expression is a universal strategy for organisms to develop methodically and adapt to various environmental conditions. However, regulation at the transcriptional level often has the disadvantage of poor timeliness. In contrast, mRNA regulation can achieve rapid and local changes in the synthesis of specific proteins in response to certain developmental cues or stress challenges, thus becoming a crucial means for cells to reprogram gene expression. In eukaryotes, a conserved mechanism has evolved in which translation inactive mRNAs can be compartmentalized by RNA-binding proteins (RBPs) to visible membraneless cytoplasmic structures generally termed messenger ribonucleoprotein (mRNP) granules (Erickson and Lykke-Andersen, 2011). Evidence is currently mounting that mRNP granules are assembled *via* a physical process named liquid-liquid phase separation (LLPS) and exhibit liquid-like physical properties (Alberti et al., 2019).

Processing bodies (PBs) and stress granules (SGs) are two well-studied mRNP granules that are widely present in eukaryotes during various stressful conditions (Erickson and Lykke-Andersen, 2011). Although these two cytoplasmic foci are considered to be distinct organelles, they are able to interact transiently (Buchan et al., 2008; Wilbertz et al., 2019), and both are highly dynamic aggregates in which the mRNAs and proteins are exchanged frequently with the cytoplasm and between each other (Anderson and Kedersha, 2006). PBs play a dual role in mRNA decay and storage, depending on mRNAs and context (Decker and Parker, 2012; Aizer et al., 2014). Similarly, SGs are also thought to function in translational repression by storing mRNAs and translation factors (Buchan and Parker, 2009). Consistent with the fact that PBs and SGs in yeast and mammalian cells are functionally linked, they are found to share some protein components, and both harbor translation repressors and proteins related to RNA turnover (Guzikowski et al., 2019). RNA is another major group recruited into mRNP granules. A surge of recent studies have reported that about 10–20% of bulk cytoplasmic RNA can localize to mRNP granules in both yeast and mammals, most (∼80%) of which are mRNAs (Hubstenberger et al., 2017; Namkoong et al., 2018; Wang et al., 2018). Depending on the cellular conditions, mRNAs stored in PBs and SGs can either reenter the normal translation cycle (Brengues et al., 2005; Merret et al., 2017; Jang et al., 2019), or be degraded through the RNA decay pathway (Sheth and Parker, 2003).

Plants sense developmental cues and continue to form new tissues and organs, which are different from animals. Moreover, as sessile organisms, plants are often subjected to various environmental constraints. These characteristics suggest that plants may rely more on mRNA regulation. Recently, structures similar to mRNP granules have been continuously observed in plants (Pomeranz M. C. et al., 2010; Motomura et al., 2015). Studies in Arabidopsis (*Arabidopsis thaliana*) have demonstrated that plants contain genes encoding homologs of some characterized PB and SG proteins in yeast and mammals (Xu and Chua, 2011; Kosmacz et al., 2019). Intriguingly, mounting evidence shows that many plant Cysteine3Histidine (CCCH) zinc-finger proteins can be located in cytoplasmic foci resembling PBs and SGs (Pomeranz M. C. et al., 2010; Pomeranz M. et al., 2010; Jan et al., 2013).

CCCH zinc-finger proteins possess a zinc-finger motif(s) composed of separated three cysteines and one subsequent histidine (C-x_4–15_-C-x_4–6_-C-x_3_-H), and are widely distributed in eukaryotes (Wang et al., 2008). In plants, tandem CCCH zinc-finger (TZF) proteins with two zinc-finger motifs usually account for the majority (Wang et al., 2008). In Arabidopsis, there are 11 TZF proteins containing an arginine-rich region (RR) followed by a plant-unique TZF motif, named AtTZF1-AtTZF11 (Pomeranz M. C. et al., 2010). Functional studies in Arabidopsis and other plants revealed that CCCH zinc-finger genes play critical regulator roles in many aspects of plant growth and development, hormone responses and environmental responses (Bogamuwa and Jang, 2014). Since the single KO mutants of these genes often exhibited subtle or no phenotypes, overexpression was often used to characterize the functions of these genes, and it has been found that moderately overexpressed transgenic plants generally exhibit enhanced tolerance to certain stresses (Lin et al., 2011; Bogamuwa and Jang, 2014). Although most CCCH zinc-finger genes diverse in expression patterns and functions, all RR-TZFs and another two TZFs (AtC3H14 and AtC3H15) in Arabidopsis and rice (*Sativa Oryza*) OsTZF1 can localize to cytoplasmic foci in protoplasts (Pomeranz M. et al., 2010; Jan et al., 2013). Moreover, the co-localization of Arabidopsis AtTZF1/4/5/6/9 and rice OsTZF1 with the marker proteins of PBs and SGs has also been verified (Pomeranz M. C. et al., 2010; Bogamuwa and Jang, 2013; Jan et al., 2013; Maldonado-Bonilla et al., 2014). Previously, we found that AtC3H18-Like (At3g52980) is the only non-TZF protein that has been identified to show cytoplasmic foci localization (Xu et al., 2020a). The similarity in the subcellular localization pattern implies that these CCCH zinc-finger proteins may share a general molecular mechanism to achieve their biological functions. However, the connection between the specific localization of these proteins and their functions still remains largely elusive.

The development of haploid male gametophyte (or pollen) of flowering plants is a delicate process involving single-cell ontogeny and two-cell lineages’ cooperation to enable double fertilization, making pollen a fascinating cell system in which to study gene expression and regulation. Since pollen development requires the orderly expression and regulation of thousands of genes in a short period of time (Honys and Twell, 2004), it will be intriguing to investigate whether there are mRNP granules involved in mRNA regulation during male gametogenesis. Here, we report that the overexpression of AtC3H18, another non-TZF protein, can promote the formation of AtC3H18-positive granules in pollen in a dose-dependent manner, leading to different male sterility phenotypes. We also demonstrate that AtC3H18 can co-localize with PB and SG markers when transiently expressed in tobacco epidermal cells. Moreover, we show that AtC3H18-positive granules can exhibit liquid-like characteristics and that their assembly process is highly dynamic and depends on the availability of mRNAs. Thus, we conclude that AtC3H18-positive granules are typical mRNP granules and speculate that AtC3H18 may participates in microgametogenesis by acting as a key component of pollen mRNP granules.

## Materials and Methods

### Plant Materials and Growth Conditions

*Arabidopsis thaliana* Columbia-0 plants were grown on nutrient soil in a phytotron (20 ± 2°C, 16-h-light/8-h-dark). Seven-day-old seedlings grown on synthetic Murashige and Skoog (MS) medium containing 2% sucrose and 0.9% plant agar at 22°C under a 14-h-light/10-h-dark regime. Tobacco plants were grown in the growth room (26 ± 2°C, 16-h-light/8-h-dark).

### Molecular Cloning and Vector Construction

All vectors were constructed with restriction enzyme digestion and ligation or with recombinant methods using a ClonExpress II One-Step Cloning Kit (Vazyme Biotech). The promoter region of *AtC3H18* and the coding regions of *AtC3H18*, *DCP2* and *PABP8* were amplified from genomic DNA and cDNA. A 2,198 bp fragment upstream of the ATG codon of *AtC3H18* were cloned into *pBI101* vector (Clontech) flanking *GUS* reporter gene (*Pro_*AtC*3*H*18_:GUS*) or the reconstructed *pBI101*, which *GUS* was replaced by *GFP* (*Pro_*AtC*3*H*18_:GFP*). The coding region of *AtC3H18* was inserted upstream of *GFP* in *Pro_*AtC*3*H*18_:GFP* plasmid to generate overexpression constructs. For tobacco transient expression, the coding regions of genes or fragments were transferred to *pFGC-GFP* or the reconstructed vector *pFGC-RFP* to achieve the high plasmid yield in *E.coli* and enhance expression in tobacco leaves. CRISPR-Cas9 technology was used to generate *atc3h18* mutants. Three targets within *AtC3H18* were designed. For each target site, two complementary 25-bp oligonucleotides with a 20-bp target sequence (Supplementary Table 1) were synthesized. Each oligo pairs were cloned into the *Bbs*I site of AtU6-26SK vector. Then, these three sgRNA expression cassettes were concatenated in tandem and cloned into the *pBI121* vector containing the hSpCas9 expression cassette. Primers are listed in Supplementary Table 1.

### Transient and Stable Plant Transformations

Transient expression experiments were conducted on leaves of 4-week-old tobacco plants by using infiltrated method. Fluorescent signals were analyzed by using a laser confocal scanning microscope (Nikon, A1; termed thereafter A1 microscope) after 48 h of infection. Images were taken with the NIS-elements AR software (Nikon) using ND Acquisition with or without Z Movement. Quantitative analysis of colocalization was conducted by using the “Colocalization Finder” tool in ImageJ.

For stable transformation, a standard floral dipping method was used. T_1_ kanamycin-resistant transgenic plants were selected on MS/agar medium containing 50 mg⋅L^–1^ kanamycin. T_2_ and T_3_ transgenic plants containing *Pro_*AtC*3*H*18_:GUS* were visualized by staining to determine the GUS activity. T_2_ and T_3_ transgenic plants containing pollen with GFP fluorescence were identified by using a fluorescent microscope (Nikon, ECLIPSE 90i). The *atc3h18* homozygous mutants were identified by PCR and sequencing. RNA extraction and qRT-PCR were performed as described by Xu et al. (2020a). *TUB4* was selected as reference gene to normalize the quantity of total RNA. All primers used are listed in Supplementary Table 1.

### Phenotypic and Cytological Observation

Alexander and DAPI solutions were used to investigate pollen viability and the development of pollen nuclei, respectively. GFP fluorescence micrographs and Alexander staining micrographs of anthers were captured by using a fluorescent microscope (Nikon, ECLIPSE 90i). Pollen fluorescence (GFP and DAPI) micrographs were captured by using an A1 microscope. Scanning electron microscopy (SEM), semi-thin section microscopy and transmission electron microscopy (TEM) analysis were performed as described by Lin et al. (2014) with some modifications.

### Quantification of Fluorescence and Granules in AtC3H18-GFP Transgenic Pollen and Roots

GFP fluorescence in *Pro_*AtC*3*H*18_:AtC3H18-GFP* pollen was quantified as described by Borg et al. (2011) with some modifications. Five representative T_3_ plants generated by three T_1_ lines were analyzed by microscopy. Pooled pollen samples were constructed by mixing bicellular and tricellular pollen from at least 10 floral buds for each plant. The A1 microscope with a Plan Apo 40×/1.25 WI water immersion objective was used to analyze the fluorescence of pooled pollen. The parameters during image capture of all samples remained constant. For each selected pollen, a merged image (6-slide stack of ∼2.00 μm thickness) was processed with “Measure” tool in ImageJ to determine the mean fluorescence intensity and area of whole pollen. Each image from single cutting plane was analyzed using “3D Objects Counter” tool to calculate the number of granules (larger than 25 voxels). The statistical granule number of each pollen was determined by adding the number of granules in the six images.

Heat treatment and CHX treatment on the roots of seven-d-old transgenic seedlings were carried out as described by Motomura et al. (2015) with some modifications. The fluorescence in cells of young roots was observed by using ND Acquisition with Z Movement from the A1 microscope. Images were analyzed with ImageJ. A *t*-test was conducted to analyze experimental results in which the two conditions were compared.

### Fluorescence Recovery After Photobleaching

Fluorescence recovery after photobleaching (FRAP) was performed on tobacco leaves transiently expressing GFP-AtC3H18 by using an A1 microscope with a Plan Apo 40×/1.25WI water immersion objective. Droplet-sized spots were bleached using a 405 nm laser with 10% intensity, and the changes in fluorescence intensity over time was collected with a 488 nm laser. Data of ROIs were obtained from the NIS-elements AR software.

## Results

### *AtC3H18* Encodes a Non-tandem Zinc-Finger Protein That Is Highly Expressed in Pollen

**FIGURE 1:**
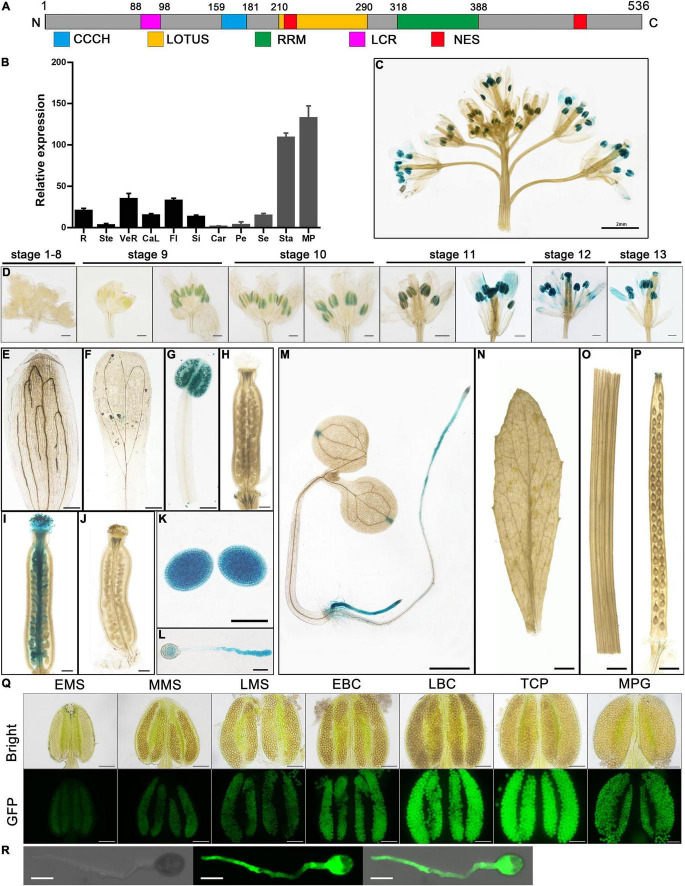
Temporal and spatial expression pattern of *AtC3H18*. **(A)** Structure of *AtC3H18* encoded protein. CCCH, CCCH zinc-finger motif; LOTUS, Limkain, Oskar, and TUdor-containing proteins 5 and 7; RRM, RNA-recognition motif; LCR, low complexity region; NES, Nuclear export signal. **(B)** Relative expression analysis showed that *AtC3H18* is highly expressed in pollen. The data was downloaded from *Arabidopsis* eFP Browser. Values are mean ± *SD*. R, root; Ste, stem; VeR, vegetative rosette; CaL, cauline leaf; Fl, flower stage 12; Si, seeds stage 5 w/siliques; Car, carpels at Fl; Pe, petals at Fl; Se, sepals at Fl; Sta, stamens at Fl; MP, mature pollen. GUS staining of *Pro_*AtC*3*H*18_:GUS* transgenic plants in inflorescence **(C)**, floral buds **(D)**, sepal **(E)**, petal **(F)**, mature anther **(G)**, mature unfertilized pistil **(H)**, wild-type pistil hand-pollinated with *Pro_*AtC*3*H*18_:GUS* pollen **(I)**, *Pro_*AtC*3*H*18_:GUS* pistil hand-pollinated with wild-type pollen **(J)**, mature pollen grains **(K)**, germinated pollen **(L)**, 7-d-old seedling **(M)**, cauline leaf **(N)**, stem **(O)** and silique **(P)**. **(Q)** GFP fluorescence intensity of *Pro_*AtC*3*H*18_:GFP* transgenic anthers. **(R)** GFP fluorescence signal in germinated pollen. EMS, MMS, LMS, early, mid, late microspore; EBP, LBP, early, late bicellular pollen; TCP, tricellular pollen; MPG, mature pollen grain. Bars = 2 mm in **(C)**, 200 μm at stage 9 and stage 10 in **(D)**, **(E)** to **(J)**, 500 μm at stage 11 to stage 13 in **(D)**, 20 μm in **(K)** and **(L)**, 1 mm in **(M)** to **(P)**, 100 μm in **(Q)**, and 10 μm in **(R)**.

*AtC3H18* encodes a non-TZF protein of 536 amino acids with a C-X_7_-C-X_5_-C-X_3_-H zinc-finger motif, two putative RNA-binding domains (RBDs, LOTUS domain and RRM domain), and two Nuclear Export Signals (NES) (Figure 1A). In Arabidopsis, a total of eleven proteins contain both CCCH motif and RRM domain, including AtC3H18 (Supplementary Figure 1A). Eight putative paralogs of AtC3H18 were identified by BLASTP analysis, but their overall amino acid sequence identities were relatively low (9–55%, Supplementary Figures 1B,C). AtC3H18 is most closely phylogenetically related to AtC3H18-Like (AtC3H18L), another non-TZF protein that has been identified by our lab previously (Xu et al., 2020a).

The microarray data of *AtC3H18* from *Arabidopsis* eFP Browser shows that *AtC3H18* is highly expressed in stamens, especially in mature pollen (Figure 1B). Here, we analyzed the temporal-spatial expression pattern of *AtC3H18* in detail. A *Pro_*AtC*3*H*18_:GUS* (β-glucuronidase) reporter system was used to monitor the *AtC3H18* expression during floral development. GUS signals in anthers appeared at stage 9, reached maximum at late stage 11 and stage 12, and declined slightly at stage 13 (Figures 1C–H). GUS activity could also be detected in germinated pollen tubes *in vivo* and *in vitro* (Figures 1I–L). Additionally, roots and the apex of cotyledons of 7-d-old seedlings showed obvious GUS activities (Figure 1M). However, no signal was found in leaves, stems, and siliques (Figures 1N–P).

To avoid the contamination of surrounding tissues by GUS histochemical staining, we also generated *Pro_*AtC*3*H*18_:GFP* transgenic plants. The results showed that *AtC3H18* began to express in early microspores and peaked in late bicellular pollen and tricellular pollen. Except for the developing pollen, no obvious GFP signal was detected in other structures of anthers (Figure 1Q and Supplementary Figure 2A). Strong fluorescence was also observed in pollen tubes when germinated *in vitro* (Figure 1R). As a control, only a weak autoflurescence signal was observed in wild-type anthers and pollen (Supplementary Figures 2B,C). These results suggest that *AtC3H18* is predominantly expressed in pollen at the late stage of pollen development, indicating that it may be involved in microgametogenesis.

### Overexpression of *AtC3H18* Results in Reduced Male Fertility

We designed three targets within *AtC3H18* genome using CRISPR/Cas9 technology, and obtained three ideal homozygous mutants (named *atc3h18-ko-21*, *atc3h18-ko-30* and *atc3h18-ko-40*, respectively) with large genomic fragment deletion of *AtC3H18* (Supplementary Figure 3). However, no obvious developmental defects were observed in mutants. During reproductive development stage, all mutants could produce a large amount of fertile pollen grains (Supplementary Figure 4), indicating that there may be genes (e.g., *AtC3H18L*) redundant with *AtC3H18*.

**FIGURE 2:**
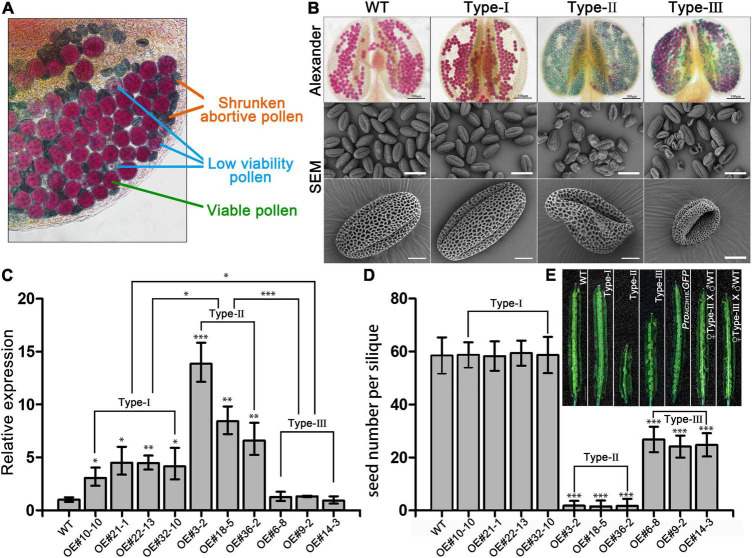
Overexpression of AtC3H18 results in three types of pollen phenotypes. **(A)** Alexander staining of a representative anther from a T_1_ line of *AtC3H18* overexpression transgenic plants. **(B)** Alexander staining of anthers and scanning electron microscopy (SEM) of pollen grains produced by three types of *AtC3H18* overexpression transgenic plants named Type-I, Type-II and Type-III, respectively. **(C)** qRT-PCR analysis of *AtC3H18* in inflorescences of WT, Type-I, Type-II, and Type-III transgenic plants. *TUB4* was used as the normalization control, and the expression of *AtC3H18* in WT was set as 1. Values are mean ± SD. **(D)** Seed number per silique. Error bars represent SD, n = 30. WT, wild-type; Type-I/II/III, Type-I/II/III *Pro_*AtC*3*H*18_:AtC3H18-GFP* transgenic plants; *Pro_*AtC*3*H*18_:GFP*, *Pro_*AtC*3*H*18_:GFP* control line; ♀Type-II/III X ♂. WT, emasculated flowers of Type-II/III plants cross-pollinated with wild-type pollen. Asterisks on columns indicate statistically significant differences from the WT calculated using Student’s *t*-test: *, *P* ≤ 0.05; ^**^, *P* ≤ 0.01; and ^***^, *P* ≤ 0.001. **(E)** Representative images of fully development siliques. Bar = 2 mm.

Gain-of-function mutagenesis is an excellent approach allows the characterization of functionally redundant genes. Therefore, we further generated Arabidopsis overexpressing lines to investigate the role of *AtC3H18* during pollen development. Thirty-six *Pro_*AtC*3*H*18_:AtC3H18-GFP* transgenic Arabidopsis plants were obtained that showed similar phenotypes. Alexander staining revealed that the anthers from all these T_1_ transgenic plants contained varying amounts of viable pollen, pollen with low viability but normal size, and shrunken abortive pollen (Figure 2A). Further observations indicated that the phenotypes of the progenies of these transgenic plants can be divided into three types (Figure 2B): Type-I, homozygous plants that generate a large number of fertile pollen grains that could hardly be distinguished from wild-type; Type-II, homozygous plants that produce normal-sized but non-viable or low viability pollen with severe collapse; and Type-III, heterozygous plants that yield half of fertile pollen and half of completely aborted and severely shrunken pollen. By contrast, all the *Pro_*AtC*3*H*18_:GFP* transgenic plants did not show any growth defects both at vegetative and reproductive growth stages (Figure 1Q and Supplementary Figure 5).

For each type of transgenic plants, the progeny of at least three lines were selected for further study. The expression level of *AtC3H18* in Type-II floral buds increased by 5- to 15-fold, which was significantly higher than Type-I (2- to 5-fold) and Type-III (did not show a significant increase) (Figure 2C). All the transgenic plants did not show any abnormal phenotype except siliques and stamens (Supplementary Figure 5). In comparison with the wild-type, the siliques from self-pollinated Type-I plants had a similar number of seeds, whereas Type-II and Type-III had fewer seeds (Figures 2D,E). Moreover, the F1 seeds developed normally when the emasculated flowers of Type-II and Type-III plants were cross-pollinated with wild-type pollen (Figure 2E), indicating that the reduced seed set of self-pollinated Type-II and Type-III plants was due to the male transmission defect caused by the reduced male fertility.

### Pollen Abortion of Type-II and Type-III Plants Begins at Different Pollen Developmental Stages

**FIGURE 3:**
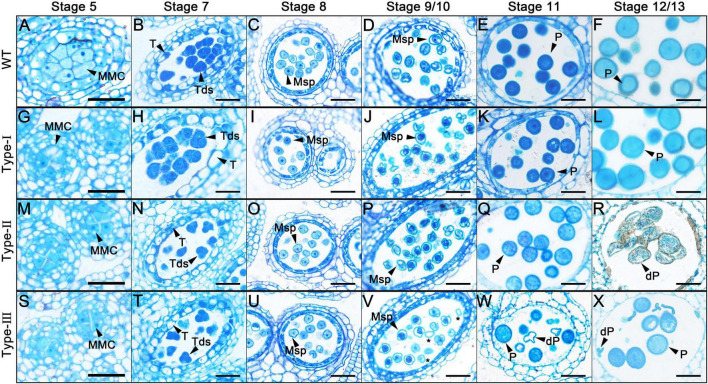
Semi-thin transverse sections of *AtC3H18* overexpression transgenic anthers. Semi-thin sections of anthers from the wild-type **(A–F)**, Type-I **(G–L)**, Type-II **(M–R)** and Type-III **(S–X)** of *Pro_*AtC*3*H*18_:AtC3H18-GFP* transgenic plants at anther development stage 5 **(A,G,M,S)**, stage 7 **(B,H,N,T)**, stage 8 **(C,I,O,U)**, stage9/10 **(D,J,P,V)**, stage 11 **(E,K,Q,W)**, and stage 12/13 **(F,L,R,X)**. Asterisks in **(V)** indicate microspores with less cytoplasm. MMC, microspore mother cell; Msp, microspore; P, pollen; dP, degraded pollen; T, tapetum; Tds, tetrads. Bars = 25 μm.

To determine the precise stage during which pollen abortion starts due to the overexpression of *AtC3H18*, another development in the different types of overexpression transgenic plants was investigated by light microscopy. No difference between wild-type and Type-I was observed during the whole process of pollen development (Figures 3A–L). However, detectable differences between wild-type and Type-II were first observed until stage 11 during which the vacuolated microspores finished the first mitotic division (Figures 3M–R). The cytoplasm of Type-II pollen at stage 11 appeared less stained and showed more inhomogeneous than wild-type (Figure 3Q). By stage 12/13, these pollen grains were crowded together and atrophied in the anther (Figure 3R). In anthers of Type-III transgenic plants, visible abnormalities were first observed at stage 9/10 (Figures 3S–X). About half of microspores seemed to possess less cytoplasm and were slightly stained (Figure 3V, asterisks). The cytoplasm of these pollen grains continued to degenerate until aborted (Figures 3W,X). Nevertheless, the development and degradation of the tapetum in Type-II and Type-III anthers appeared to be normal.

**FIGURE 4:**
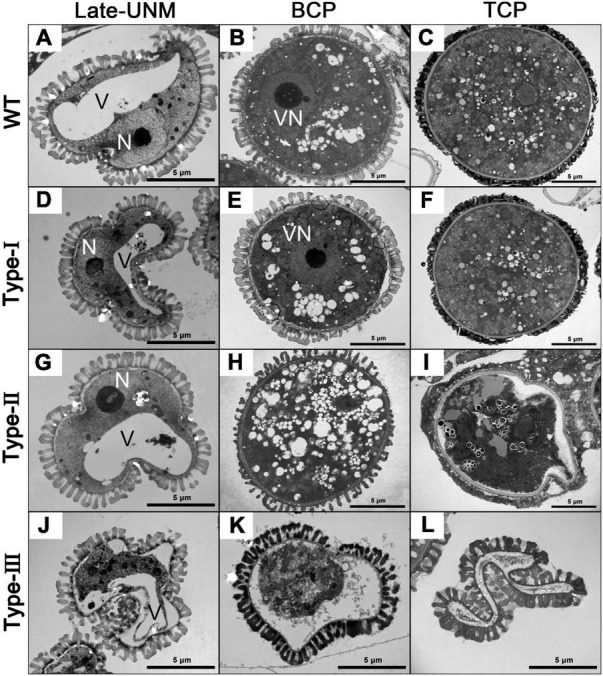
Transmission electron microscopy (TEM) of different types of *AtC3H18* overexpression transgenic pollen. Ultrastructure of microspores at different developmental stages from the wild-type **(A–C)**, Type-I **(D–F)**, Type-II **(G–I)**, and Type-III **(J–L)** of *Pro_*AtC*3*H*18_*:*AtC3H18-GFP* transgenic plants. Late-UNM, late uninucleate microspore; BCP, bicellular pollen; TCP, tricellular pollen; N, nucleus; V, Vacuole; VN, vegetative nucleus. Bars = 5 μm.

Pollen ultra-structures were further observed by TEM (Figure 4). As expected, no difference was observed between Type-I and wild-type pollen (Figures 4A–F). By the late uninucleate stage, Type-II microspores were also not detectably different from the wild-type (Figure 4D). However, Type-III transgenic microspores were irregular in shape, and the cytoplasm began to degrade and detach from the cell wall (Figure 4J). At the bicellular stage, a large number of small vacuoles were distributed throughout the cytoplasm of Type-II pollen (Figure 4H). In Type-III bicellular pollen, the cellular structures and contents were further degraded and disappeared (Figure 4K). By the tricellular stage, both wild-type and Type-I pollen had well-developed dense cytoplasm and various organelles, whereas Type-II pollen began to shrink, leaving some structures that were no longer distinguishable (Figure 4I). More seriously, Type-III tricellular pollen grains have completely collapsed without any cellular content (Figure 4L). Despite the degradation of pollen contents, the pollen wall development of Type-II and Type-III transgenic pollen remained basically normal. Together, these results indicate that the abnormal development of Type-II pollen begins at the binucleate stage, while in Type-III transgenic plants, it starts from the late-uninucleate stage, which is earlier than that in Type-II plants.

### The Continuous Formation of Numerous AtC3H18-Positive Granules Impairs Pollen Development

Why does the overexpression of *AtC3H18* result in different pollen phenotypes? Is this only related to the level of overexpression? Since the qRT-PCR results cannot accurately predict the expression level of *AtC3H18* in each pollen at each stage, we used GFP that translationally fused to AtC3H18 to make it possible to visually observe the fusion protein in each pollen.

**FIGURE 5:**
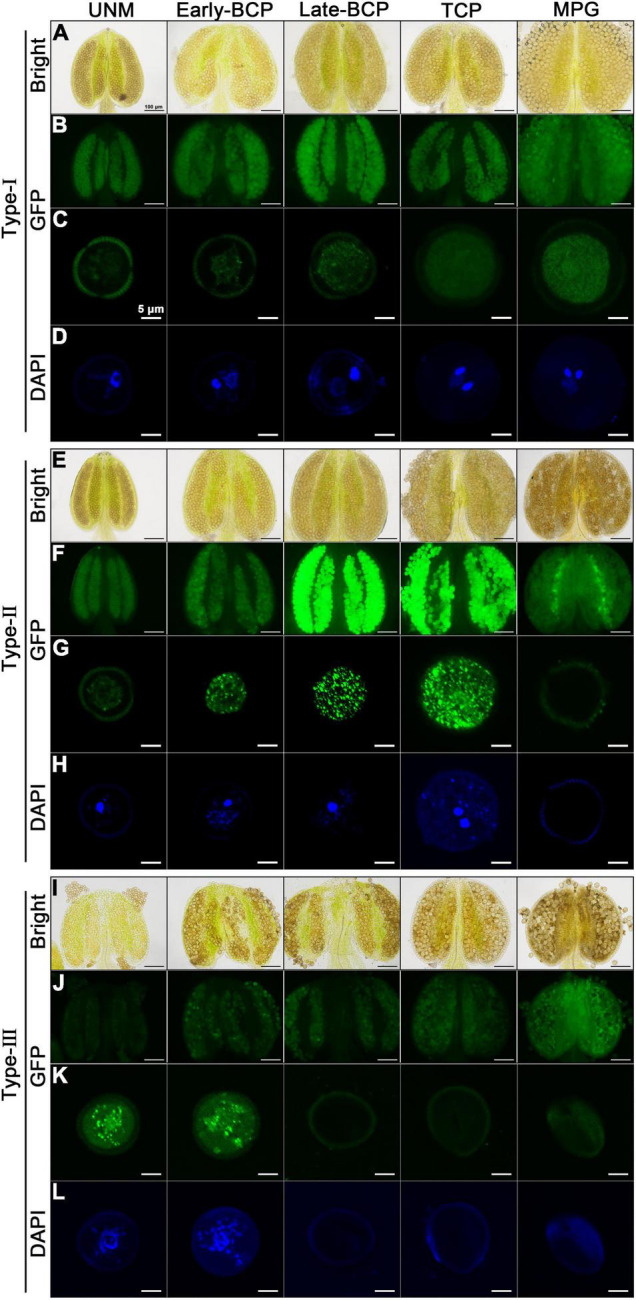
Observation of pollen nucleus development and AtC3H18-GFP fusion protein in anthers and pollen grains of different types of *AtC3H18* overexpression transgenic plants. Anthers and pollen grains from different developmental stages of Type-I **(A–D)**, Type-II **(E–H)**, and Type-III **(I–L)** of *Pro_*AtC*3*H*18_*:*AtC3H18-GFP* transgenic plants were displayed. GFP images of pollen showed the formation of AtC3H18-positive granules **(C,G,K)**. DAPI staining labeled pollen nuclei **(D,H,L)**. Images in **(C,D,G,H,K,L)** were merged from multiple cutting planes. UNM, uninucleate microspores; Early/Late-BCP, early/late-bicellular pollen; TCP, immature tricellular pollen; MPG, mature pollen grain. Bars = 100 μm in Bright and GFP images of anthers, 5 μm in GFP and DAPI staining images of pollen.

Anthers and pollens from Type-I, Type-II and Type-III transgenic plants were examined (Figure 5). As expected, Type-I anthers developed normally, and pollens inside were always morphologically normal and can develop into mature pollen with high viability. The pollen nuclei were also well-developed. AtC3H18-GFP fusion protein seemed to be unevenly distributed in both uninucleate microspore (UNM) and bicellular pollen (BCP), but only a small number of tiny granules could be observed in BCP. In tricellular pollen (TCP) and mature pollen grain (MPG), the distribution of fusion protein in the cytoplasm presented a near uniform state (Figures 5A–D). Type-II anthers appeared to be normal until the MPG stage, during which the pollen grains shrunk significantly. Strikingly, a large number of AtC3H18-positive granules were detected in early/late-BCP and TCP. Coincidentally, abnormal scattered vegetative nucleus was first observed in early-BCP, which was subsequently degraded. Generative nuclei seemed to be normal, but sperm nuclei disappeared in MPG. Finally, only collapsed pollens were remained (Figures 5E–H). In Type-III transgenic pollen, AtC3H18-GFP fusion protein began to be recruited into granules at uninuclear stage. Simultaneously, we found that these microspores failed to undergo pollen mitosis I, only remained a scattered nucleus-like structure in early-BCP, which was completely disappeared at late-bicellular stage. Thereafter, these pollen grains crumpled and eventually aborted (Figure 5I–L). In brief, in Type-II and Type-III transgenic pollen, the appearance of a large number of AtC3H18-positive granules was always accompanied with abnormal pollen development and was highly consistent in time, which indicates that the pollen abortion is caused by the continuous formation of these granules.

It is worth mentioning that all pollen grains in Type-I plants are transgenic with normal development (Figures 5B,C), and the expression of *AtC3H18* in Type-II plants can also be detected before the MPG stage (Figures 5F,G). However, in Type-III plants, half of pollen is wild-type without overexpression, and the other half is transgenic pollen that can express *AtC3H18* only before early-BCP stage (Figures 5J,K). Therefore, it is reasonable and understandable that the expression of *AtC3H18* in the Type-III plant floral buds was lowest by qRT-PCR (Figure 2C). Interestingly, when comparing the GFP fluorescence signals in a single pollen at the same developmental stage, it can be inferred that the expression level of *AtC3H18* in Type-III transgenic UNM and early-BCP are very likely to be higher than that in Type-I and Type-II (Figures 5C,G,K), suggesting that the overexpression activity of *AtC3H18* in transgenic pollen may be ranked as follows: Type-III > Type-II > Type-I.

**FIGURE 6:**
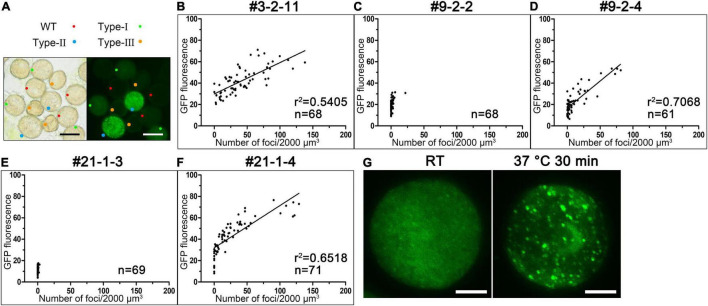
The elevated expression of AtC3H18 can promote the formation of AtC3H18-positive granules in pollen. **(A)** Anthers from T_1_ lines of *AtC3H18* overexpression transgenic plants usually contain wild-type pollen, Type-I, Type-II and Type-III transgenic pollen, simultaneously. Bars = 20 μm. **(B–F)** The scatter plots showed the relationships between the number of AtC3H18-positive granules and the fluorescence intensity of transgenic pollen produced by the T_3_ lines of *AtC3H18* overexpression transgenic plants, named OE#3-2-11 **(B)**, OE#9-2-2 **(C)**, OE#9-2-4 **(D)**, OE#21-1-3 **(E)**, and OE#21-1-4 **(F)**, respectively. Each point represented an individual pollen, and at least 60 pollen were analyzed for each plants. Lines in OE#3-2-11 **(B)**, OE#9-2-4 **(D)** and OE#21-1-4 **(F)** depicted the degree of linear regression. **(G)** A heat shock of 37°C for 30 min promotes the formation of large AtC3H18-positive granules. Bars = 5 μm.

We also examined the fluorescent signals in a large number of pollen grains produced by 36 T_1_ lines of *Pro_*AtC*3*H*18_:AtC3H18-GFP* transgenic plants. The results showed that each anther at TCP stage of all lines simultaneous harbored wild-type pollen, transgenic pollen containing fusion protein with dispersion positioning (Type-I) or granule positioning (Type-II), and aborted pollen (Type-III) (Figure 6A). This observation was also consistent with the results of Alexander staining showing that each anther contained pollen grains with distinct phenotypes (Figure 2A). Furthermore, we found that the assembly of granules was closely related to the expression level of AtC3H18-GFP fusion protein. Thus, we systematically analyzed pollen samples (mainly bicellular and tricellular pollen) from five representative T_3_ plants generated by three T_1_ lines. The results demonstrated that the pollen with moderate overexpression of *AtC3H18* (OE#9-2-2 and OE#21-1-3, and some pollen of OE#9-2-4 and OE#21-1-4) had few detectable granules (Figures 6C–F). Remarkably, when AtC3H18 was strongly overexpressed (OE#3-2-11, and some pollen of OE#9-2-4 and OE#21-1-4), a large number of granules could be detected. Moreover, the number of granules was positively correlated with the intensity of fluorescence (Figures 6B,D,F). In contrast, pollen expressing free-GFP never formed fluorescent puncta similar to AtC3H18-positive granules (Supplementary Figure 1A). Interestingly, a heat shock of 37°C for 30 min could also make granules larger and more visible (Figure 6G). These results manifested that the assembly of AtC3H18-positive granules in pollen could be promoted by elevated expression of AtC3H18 and heat stress.

### AtC3H18 Can Co-localize With Processing Body and Stress Granule Markers

The punctate distribution of the fusion protein in pollen mentioned above indicated that AtC3H18 may exhibit similar subcellular localization pattern like AtTZFs. To verify this speculation, we first analyzed the subcellular localization of AtC3H18 by expressing fusion protein GFP-AtC3H18 transiently in leaf epidermal cells of H2B-RFP transgenic tobacco (*Nicotiana tabacum*) plants. Interestingly, although the fusion protein was driven by the high-expression promoter *CaMV35S*, GFP-AtC3H18 was always diffusely distributed in the cytoplasm at room temperature (RT). However, heat treatment can induce the aggregation of fusion protein and form cytoplasmic foci, resembling PBs and SGs (Supplementary Figure 6).

**FIGURE 7:**
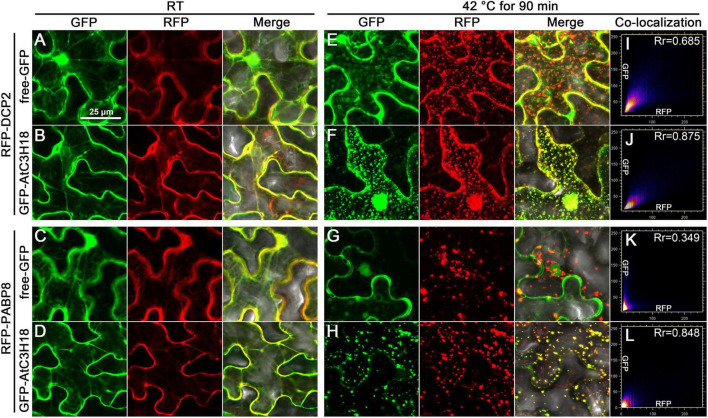
AtC3H18 can co-localize with PB and SG marker proteins during heat stress. Co-localization images of free-GFP and GFP-AtC3H18 fusion protein with RFP-DCP2 **(A,B,E,F)** or RFP-PABP8 **(C,D,G,H)** in *N. benthamiana* leaf epidermal cells at room temperature (RT, **A–D**) and after heat treatment at 42°C for 90 min **(E–H)**, respectively. Pictures represent epifluorescence (GFP and RFP) and merged images (Merge). All the images shown here are the magnified images of the areas depicted by the white frames in Supplementary Figure 7. All Images were merged from multiple cutting planes. Bars = 25 μm. **(I–L)**, Quantitative analysis of co-localization.

Next, co-localization experiments were implemented by using *Arabidopsis* PB and SG markers, DCP2 and PABP8 (Xu et al., 2006; Anderson and Kedersha, 2008). When GFP-AtC3H18 was co-expressed with RFP-DCP2 or RFP-PABP8 (Supplementary Figure 7A) in tobacco leaves, all fusion proteins were mainly dispersed in the cytoplasm at RT (Figures 7A–D and Supplementary Figures 7B–E). As expected, heat treatment induced the formation of cytoplasmic foci, resulting in good co-localization of AtC3H18 with DCP2 or PABP8 in the granular structures (Figures 7F,H,J,L and Supplementary Figures 7G,I). As a negative control, no co-localization of free-GFP with DCP2 or PABP8 was observed in the granular structures (Figures 7E,G,I,K and Supplementary Figures 7F,H). Similar pattern was also observed when GFP was translationally fused to the C-terminus of AtC3H18 (Supplementary Figures 7J–M). These results demonstrate that AtC3H18 has a specific subcellular localization pattern, i.e., mainly diffused in the cytoplasm at RT, but can be recruited into cytoplasmic foci during heat stress and co-localize with PB and SG markers.

### Highly Dynamic Properties of AtC3H18-Positive Granules

**FIGURE 8:**
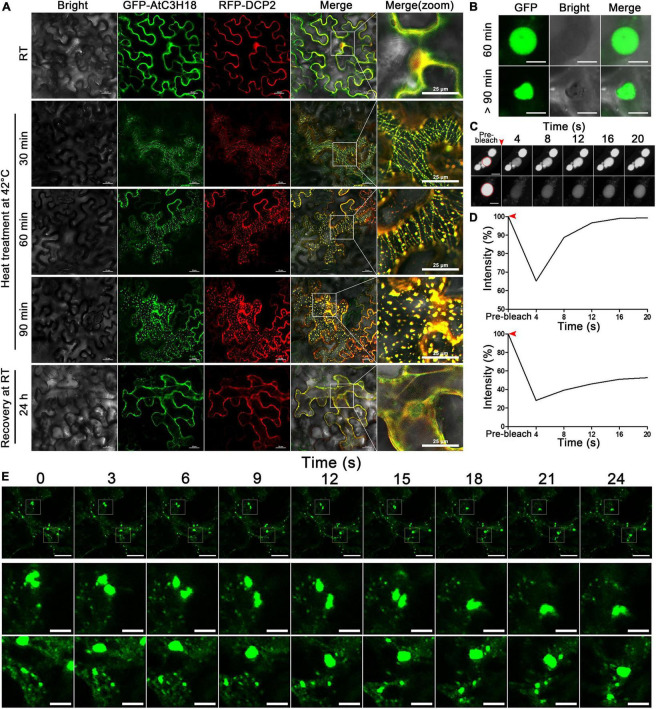
The formation process and the highly dynamic nature of AtC3H18-positive granules. **(A)** The highly dynamic formation of AtC3H18-positive granules during heat stress. Pictures represent white field images (Bright), epifluorescence (GFP-AtC3H18 and RFP-DCP2), merged images (Merge), and magnified images in Merge [Merge (zoom)]. Bars = 25 μm. **(B)** Shapes of AtC3H18-positive granules in *N. benthamiana* leaf epidermal cells during heat treatment. Bars = 5 μm. **(C)** FRAP images showed fluorescence recovery of two representative AtC3H18-positive granules at different time points *in vivo*. Red arrowhead marks photobleaching events, red circles represent the bleaching areas. Bars = 5 μm. **(D)** FRAP recovery curves of two representative AtC3H18-positive granules in **(C)**. Red arrowheads mark photobleaching events. **(E)** Montages of AtC3H18-positive granules deforming and fusing during heat treatment. The next two rows of images are the enlargements of the first row. Bars = 20 μm in the first line and 5 μm in the second and third lines. All Images were captured from a single cutting plane.

We then monitored the formation of AtC3H18-positive granules in tobacco leaf epidermal cells over time during heat stress (Figure 8A). After about 30 min of heat treatment, GFP-AtC3H18 and RFP-DCP2 fusion proteins showed linear structures and highly overlapped. Noticeably, many small granules attached to linear structures began to appear and move actively. An hour later, these small granules fused together to form lager granules resembling liquid-like spherical droplets. When examined over 90 min, most of the granules changed from round to irregularly shape and became relatively stable. After 24 h of recovery at RT, no granules were observed, and fusion proteins were once again dispersed in the cytoplasm, which means that previously formed granules have been dissolved, indicating that these granules are reversible. These results suggest that AtC3H18 tends to undergo transition from a dispersion state to a liquid and then to a gel/solid state after thermal agitation, and this process is closely related to the duration of heat stress.

The dynamic nature of AtC3H18-positive granules is further reminiscent of some mRNP granules like PB and SG, which are formed *via* LLPS mechanism and show many liquid-like characteristics. Thus, we next sought to examine whether AtC3H18-positive granules also exhibited liquid properties. Encouragingly, the granules showed a nearly perfect spherical shape after mild heat treatment at 35°C for 60 min, but gradually became irregular as treatment continued (Figure 8B). FRAP experiments showed that the fluorescence of granules recovered completely or partially with a few seconds after photobleaching (Figures 8C,D), demonstrating the liquid-like mobility, and indicating that AtC3H18 can exchange with the cytoplasm quickly and frequently. Confocal time-lapse analysis also showed that AtC3H18-positive granules underwent frequent fusion and fission events in the cytoplasm (Figure 8E). These findings further suggest that AtC3H18-positive granules are a kind of mRNP granules similar to PBs and SGs.

### The Assembly of AtC3H18-Positive Granules Depends on mRNA Availability

**FIGURE 9:**
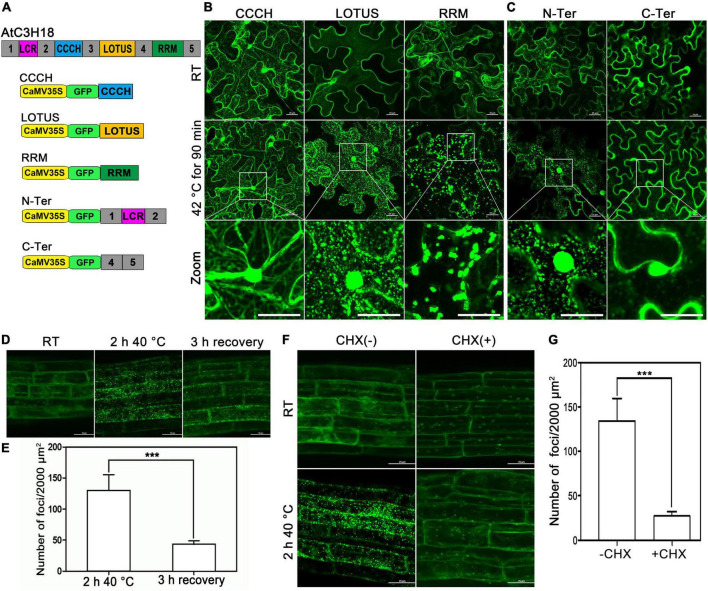
LOTUS, RRM and N-terminus are responsible for targeting AtC3H18 to the cytoplasmic foci, and mRNAs are required for the formation of AtC3H18-poisitive granules. **(A)** Schematic diagram of AtC3H18 protein structure and the constructs used for the subcellular localization analyzes of truncated AtC3H18. **(B)** The localization of CCCH zinc finger motif, LOTUS domain and RRM domain in *N. benthamiana* leaf epidermal cells at room temperature (RT) and after heat stress. **(C)** The localization of N-Terminus (N-Ter, amino acids 1 to 158) and C-Terminus (C-Ter, amino acids 291 to 317 and 389 to 536). The localization of AtC3H18 **(D)** and the quantification of granules **(E)** in young roots of *Pro_*AtC*3*H*18_:AtC3H18-GFP* transgenic seedlings after heat treatment and after 3 h recovery. The localization of AtC3H18 **(F)** and the quantification of granules **(G)** in young roots of *Pro_*AtC*3*H*18_:AtC3H18-GFP* transgenic seedlings after heat treatment with or without CHX treatment. Pictures represent at least three independent analyzes. **(E,G)**, *n* = 5 biological repeats, mean ± SD. ***P < 0.001. All Images were merged from multiple cutting planes. Bars = 25 μm.

To explore how AtC3H18 is targeted to granules, we compared the localization of GFP fused to different domains (hereafter designated as CCCH, LOTUS and RRM, respectively) and sequences (hereafter designated as N-Ter and C-Ter, respectively) of AtC3H18 (Figure 9A). At RT, all the fusion proteins were localized in the cytoplasm when transiently expressed in tobacco (Figure 9B), similar to the full-length AtC3H18. After heat treatment, LOTUS, RRM and N-Ter became associated with the foci, while the localization of CCCH and C-Ter remained cytoplasmic (Figures 9B,C). These findings manifest that AtC3H18 is targeted to granules by LOTUS domain, RRM domain and N-ter. Interestingly, the granules formed by RRM seemed to be much less in quantity but larger in size than those formed by LOTUS and N-Ter, which means that these domains or sequences may contribute differently to the assembly of AtC3H18-positive granules. However, more quantitative statistics are still needed.

Both LOTUS and RRM domains have the potential to bind RNA (Birney et al., 1994; Anantharaman et al., 2010), which indicates that mRNA may be essential for AtC3H18 targeting to granules. The *Pro_*AtC*3*H*18_:AtC3H18-GFP* transgenic plants were used to verify this hypothesis. Consistent with the results in tobacco, heat stress can also induce AtC3H18 in young roots to aggregate into granules from a dispersed state. Furthermore, most of these granules can be disassembled and re-diffuse into the cytoplasm after 3 hours of recovery at RT (Figures 9D,E). As a control, no obvious differences were observed in the roots of *CaMV35S*:*GFP* seedlings at RT or after heat treatment (Supplementary Figure 8). Cycloheximide (CHX) is a protein synthesis inhibitor that blocks the release of mRNAs from polysomal complexes. Compared with untreated samples, the number of AtC3H18-positive granules in CHX-treated roots was significantly reduced (*P* < 0.001) (Figures 9F,G), indicating that the assembly of AtC3H18-positive granules depends on the availability of mRNA, and it is further confirmed that these granules are mRNP granules encompassing certain mRNA. However, whether the assembly of AtC3H18 granules is achieved through the direct interaction of AtC3H18 with mRNA does requires more evidence.

## Discussion

The results presented here demonstrate that different overexpression levels of *AtC3H18* can result in different pollen phenotypes. By observing the process of microgametogenesis of transgenic pollens and monitoring AtC3H18-GFP fusion protein, we found that pollen abortion is associated with the formation of numerous AtC3H18-positive granules. This can be supported by two findings: (1) Type-I pollens do not form a large number of AtC3H18-positive granules and develop normally; on the contrary, Type-II and Type-III transgenic pollens contain numerous granules and are aborted. (2) In both Type-II and Type-III transgenic pollens, the massive formation of AtC3H18-positive granules is closely followed by the abnormality of pollen development. We also found that AtC3H18-positive granules are formed in a dose-dependent manner in pollen. Therefore, we conclude that high overexpression of *AtC3H18* in pollen can result in the continuous formation of AtC3H18-positive granules, which can lead to male sterility. Perhaps due to functional redundancy with another newly identified non-TZF protein named *AtC3H18L* (Xu et al., 2020a), the *atc3h18* mutants do not show any phenotype. Encouragingly, the homologous gene of *AtC3H18L* has been lost in *Brassica campestris*. Moreover, in our previous study, double mutants (*bcmf30a bcmf30c*) of *BcMF30a* and *BcMF30c*, two homologous genes of *AtC3H18* in *Brassica campestris*, showed partial male sterility (Xu et al., 2020b). These results together suggest that the appropriate expression level of *AtC3H18* in pollen is essential for the normal microgametogenesis. The construction of double mutants of *AtC3H18* and *AtC3H8L* to further verify this speculation will be one of focuses of our future work.

As far as we know, this study provided direct evidence in plants for the first time, showing that the continuous formation of cytoplasmic foci can lead to abnormal growth and development, which has been fully confirmed in mammals. The most convincing evidence is that many debilitating neurodegenerative diseases are characterized by the assembly of pathological mRNP granules and the destruction of normal mRNA metabolism (Ramaswami et al., 2013). Interestingly, overexpression of *TZFs* (e.g., *AtTZF1*, *4*, *5*, and *6*) can also induce the assembly of TZF-positive cytoplasmic foci in intact plants (Pomeranz M. C. et al., 2010; Bogamuwa and Jang, 2013). Meanwhile, studies have also found that transgenic plants with overexpression of *TZFs* often show growth defects. For instance, the overexpression transgenic plants of *AtTZF1*, *4*, *5*, and *6* all exhibited compact and crinkled leaves, and some of homozygous overexpression plants of *AtTZF1* even showed lethal phenotype (Lin et al., 2011; Bogamuwa and Jang, 2013). Constitutive overexpression of *AtC3H14* and *AtC3H15* in Arabidopsis led to dwarfing phenotypes and male sterility, respectively (Kim et al., 2014; Shi et al., 2015). However, most studies only briefly described the abnormal phenotypes without any explanation, and did not attempt to link the phenotypes with the formation of cytoplasmic foci. The findings in this study imply that the growth defects of those TZFs overexpression plants may also be caused by the persistent formation of TZFs-positive cytoplasmic foci. If this is true, it is necessary to reassess the positive functions of overexpression of TZFs that can enhance resistance to certain stresses, as this may come at the cost of normal growth and development.

We also report here that AtC3H18 is a protein component of mRNP granules, including PBs and SGs. This proposition is supported by several evidence: (1) Transiently expressed AtC3H18 can co-localize with PB and SG markers, DCP2 and PABP8, respectively, following heat stress; (2) Stably expressed AtC3H18 can be recruited into granules in intact plants both in young roots and pollen; (3) AtC3H18-positive granules exhibit many liquid-like properties, which are typical characteristic features of mRNP granules; and (4) CHX treatment inhibits the formation of AtC3H18-positive granules, indicates that they encompass certain mRNAs. A few components are critical to the structural integrity of RNP granules, commonly referred to as “scaffolds” (Banani et al., 2016; Gutierrez-Beltran et al., 2021). In contrast, the remaining majority of components, termed “clients,” are dispensable for the formation of granules, and often localize to granules only under certain circumstances (Chalupníková et al., 2008). Clients often diffuse much faster than scaffolds within granules, and exhibit rapid dynamic exchange between granules and cytoplasm (Weidtkamp-Peters et al., 2008). Here, we observed the rapid recovery of AtC3H18 after FRAP in tobacco cells, indicating that AtC3H18 plays a client role in the assembly of mRNP granules rather than a scaffold. Noticeably, at RT, AtC3H18-positive granules can be observed in pollen, but not in tobacco cells or young roots of *AtC3H18* overexpression plants. This discrepancy indicates that the critical concentration of AtC3H18 that induces the assembly of mRNP granules may vary in different cells, and that AtC3H18 acts as a promoter more than a client in this process. Recently, it was also observed that AtTZF9 (overexpression) can promote the assembly of stress-independent SGs (Tabassum et al., 2020).

The assembly and maintenance of membraneless mRNP granules are usually achieved through multivalent transient weak interactions between proteins and between proteins and RNAs (Banani et al., 2016). RBPs are vastly enriched in mRNP granules (Guzikowski et al., 2019), indicating that the interactions between RBPs and RNAs are important sources of multivalency. Intriguingly, Arabidopsis AtTZF1, AtTZF9 and AtC3H14, and rice OsTZF1, all of which are cytoplasmic focal localization proteins, can bind RNA *in vitro* (Pomeranz M. C. et al., 2010; Jan et al., 2013; Kim et al., 2014; Maldonado-Bonilla et al., 2014). Here, we showed that individual LOTUS and RRM domain of AtC3H18 can localize in cytoplasmic foci after heat stress, suggesting that the possible interactions between these two putative RBDs and their target RNAs are partially responsible for the recruitment of AtC3H18 into mRNP granules. However, it is not excluded the possibility that AtC3H18 can be recruited by the interactions between its LOTUS domain and other proteins located in mRNP granules, as studies have found that the LOTUS domains of mammalian TDRD5, TDRD7 and MARF1 can bind to proteins or complexes located in PBs (Jeske et al., 2017; Zhu et al., 2018). Studies have demonstrated that RNA identity and concentration can significantly impact the assembly and stability of mRNP granules (Zhang et al., 2015; Langdon et al., 2018). Therefore, we speculate that it might be the specificity of pollen transcriptome provides a unique protein-mRNA interactome for the assembly of AtC3H18-positive granules, so that the critical concentration of AtC3H18 forming mRNP granules in pollen is lower than that in tobacco cells.

Studies have revealed that mRNAs stored in PBs and SGs can be reused when released in time (Merret et al., 2017; Jang et al., 2019). Given that the assembly of AtC3H18-positive granules is highly dynamic and reversible (Figures 8A, 9D), we believe that mRNAs recruited in these granules may also have the potential of reentering the translation process when appropriate. However, in this study, due to the overexpression of AtC3H18, mRNP granules were continuously assembled in pollen during microgametogenesis, resulting in the inability to release the mRNAs stored in it in time. Failure to reuse mRNAs may be the direct cause of pollen abortion in Type-II and Type-III plants. Therefore, we speculate that AtC3H18 is required to be expressed at a proper level during micogametogenesis to maintain the reversibility (assembly and disassembly) of mRNP granules, so as to achieve the purpose of regulating pollen transcriptome. Encouragingly, it has been hypothesized that mRNP granules dissolved during pollen tube growth as the stored mRNA being utilized for translation and function during pollen tube growth (Hafidh and Honys, 2021). Therefore, we speculate that mRNA stored in AtC3H18-positive granules likely encodes proteins required for pollen tube growth. In addition, studies in plants and yeast have shown that ribosomal mRNAs are significantly enriched in mRNP granules (Arribere et al., 2011; Merret et al., 2017; Jang et al., 2019), combined with ribosomal mRNAs that are highly expressed in UNM and BCP (Honys and Twell, 2004), we propose another conjecture that AtC3H18 may help UNM and BCP to stabilize ribosomal mRNAs. The verification of theses hypotheses will be the focus of our future work.

## Data Availability Statement

The original contributions presented in this study are included in the article/Supplementary Material, further inquiries can be directed to the corresponding authors.

## Author Contributions

LX and JC: conceived the research. JC and YY: supervised the experiment. LX: designed and performed the experiments, analyzed the data, and prepared the figures. TL, XX, and LH: provided technical assistance. LX and YY: wrote the manuscript. All authors contributed to the article and approved the submitted version.

## Conflict of Interest

The authors declare that the research was conducted in the absence of any commercial or financial relationships that could be construed as a potential conflict of interest. The handling editor declared a past collaboration with one of the authors, LH.

## Publisher’s Note

All claims expressed in this article are solely those of the authors and do not necessarily represent those of their affiliated organizations, or those of the publisher, the editors and the reviewers. Any product that may be evaluated in this article, or claim that may be made by its manufacturer, is not guaranteed or endorsed by the publisher.
