# Supplementary material for: Highly Overexpressed AtC3H18 Impairs Microgametogenesis via Promoting the Continuous Assembly of mRNP Granules
Source: Front Plant Sci. 2022 Jul 15;13:932793. doi: 10.3389/fpls.2022.932793 (PMC9335048; doi:10.3389/fpls.2022.932793)
Supplement: Supplementary file 1 [file Data_Sheet_1.docx]

Supplementary Material

**Table S1.** List of primers.

| **Experiment** | **Primer Name** | **Sequence (5'-3')** | **Construct** | **backbone (Vector)** |
| --- | --- | --- | --- | --- |
| **AtC3H18 CDS** | AtC3H18-F | ATGAATTTCACAGAATCAATGA | - | - |
|  | AtC3H18-R | CTATGTAACCGTTGAAATCTCC |  |  |
| **AtC3H18 expression pattern** | AtC3H18pro-F1 | GACCATGATTACGCC**AAGCTT**TCAGTTTGTTCTCGGTTCG | AtC3H18pro-GUS | pBI101Δ35S |
|  | AtC3H18pro-R1 | ACAGGACGTAACATCTCTAAAAAACTTCTTATCTTAAAAA |  |  |
|  | 35S-eGFP-F | GGGACTCTAGA**GGATCC**ATGGTGAGCAAGGGCGA | CaMV35S-eGFP | pBI101ΔGUS |
|  | 35S-eGFP-R | GTTGTTGATCCCGGGCTA**GGATCC**CTTGTACAGCTCG |  |  |
|  | AtC3H18pro-F2 | GACCATGATTACGCC**AAGCTT**TCAGTTTGTTCTCGGTTCG | AtC3H18pro-eGFP | pBI101-eGFP  ΔCaMV35S |
|  | AtC3H18pro-R2 | CTTGCTCACCAT**TCTAGA**CTCTAAAAAACTTCTTATCTTAAAAA |  |  |
| **Localization** | AtC3H18-N-F | GCTGTACAAG**GGATCC**ATGAATTTCACAGAATCAATGA | eGFP-AtC3H18 | pFGC-eGFP |
|  | AtC3H18-N-R | TAATTAACTC**TCTAGA**CTATGTAACCGTTGAAATCTCC |  |  |
|  | AtC3H18-C-F | ATTTACAATTACCATGAATTTCACAGAATCAATG | AtC3H18-eGFP |  |
|  | AtC3H18-C-R | GCCCTTGCTCACCATTGTAACCGTTGAAATCTCC |  |  |
|  | RFP-F | TACAATTACCATGGTGTCTAAGGGCGAAG | pFGC-RFP | pFGCΔeGFP |
|  | RFP-R | CCTA**GGATCC**ATTAAGTTTGTGCCCCAGTT |  |  |
|  | DCP2-F | AAACTTAAT**GGATCC**ATGTCGGGCCTCCATCGA | RFP-DCP2 | pFGC-RFP |
|  | DCP2-R | GACTCACCTAAGCTGAATTACCAGATTCCA |  |  |
|  | PABP8-F | AAACTTAAT**GGATCC**ATGGCTCAGATTCAGCATCA | RFP-PABP8 |  |
|  | PABP8-R | GACTCACCTAAGGTACGATGTTGTCTCCAA |  |  |
|  | CCCH-F | GCTGTACAAG**GGATCC**GTAAAAATCTGCCATTACTTCA | eGFP-CCCH | pFGC-eGFP |
|  | CCCH-R | TAATTAACTC**TCTAGA**CTACCCGTGGAAGTACCTACAGT |  |  |
|  | LOTUS-F | GCTGTACAAG**GGATCC**GAGATCATTGAGTTACTCAAGTTAA | eGFP-LOTUS |  |
|  | LOTUS-R | TAATTAACTC**TCTAGA**CTAAACTGAATGTTGCCCATGA |  |  |
|  | RRM-F | GCTGTACAAG**GGATCC**ATTTACTTAACGTTTCCGGC | eGFP-RRM |  |
|  | RRM-R | TAATTAACTC**TCTAGA**CTACTTGACTAGAACACGTGCAT |  |  |
|  | N-Ter-F | GCTGTACAAG**GGATCC**ATGAATTTCACAGAATCAATGA | eGFP-N-Ter |  |
|  | N-Ter-R | TAATTAACTC**TCTAGA**CTACGGGAACTCGGGTAAACT |  |  |
| **AtC3H18 Over-Expression** | AtC3H18pro-F3 | GACCATGATTACGCC**AAGCTT**TCAGTTTGTTCTCGGTTCG | pBI121-AtC3H18pro | pBI121Δ35S  ΔGUS |
|  | AtC3H18pro-R3 | TTGATCCCGG**GGATCC**CTCTAAAAAACTTCTTATCTTAAAAA |  |  |
|  | AtC3H18pro-CDS-F | AGAAGTTTTTTAGAGATGAATTTCACAGAATCAATGA | pBI121-AtC3H18pro-CDS | pBI121-AtC3H18pro |
|  | AtC3H18pro-CDS-R | TGATCCCGG**GGATCC**CTATGTAACCGTTGAAATCTCC |  |  |
|  | eGFP-F | AACGGTTACAATGGTGAGCAAGGGCG | pBI121-AtC3H18pro-CDS-GFP | pBI121-AtC3H18pro-CDS |
|  | eGFP-R | TGATCCCGG**GGATCC**CTAGGATCCCTTGTACAGCTC |  |  |
| **qRT-PCR** | qTUB4-F | AAGGCTTTCCTTCATTGGTACA | - | - |
|  | qTUB4-R | CTCTCCGGCTGTAGCATCTT |  |  |
|  | qAtC3H18-F | ATTACTCCTACCTCGGCTTCT | - | - |
|  | qAtC3H18-R | AACCGTTCTGCTTGTTCCT |  |  |
| **Knockout of AtC3H18 using CRISPR-Cas9** | sgRNAa-F | *GATTG*AACGAAAGAGAAGAAGAAGA | AtU6-26-sgRNAa-SK | AtU6-26SK |
|  | sgRNAa-R | *AAAC*TCTTCTTCTTCTCTTTCGTT*C* |  |  |
|  | sgRNAb-F | *GATTG*TGACGGATAATTTTTCACCA | AtU6-26-sgRNAb-SK |  |
|  | sgRNAb-R | *AAAC*TGGTGAAAAATTATCCGTCA*C* |  |  |
|  | sgRNAc-F | *GATTG*ACAACTGTAGGTACTTCCAC | AtU6-26-sgRNAc-SK |  |
|  | sgRNAc-R | *AAAC*GTGGAAGTACCTACAGTTGT*C* |  |  |
|  | U6-b-F | GCCGCTCGAGCGTTGAACAACGGAAACTCGACT | AtU6-26-sgRNAa- AtU6-26-sgRNAb- SK | AtU6-26-sgRNAa-SK |
|  | U6-b-R | TATCGATA**CCCGGG**CTCGAGCGGCCGCCAGT |  |  |
|  | U6-c-F | GCTCGAGCCCCGTTGAACAACGGAAACTCGACT | AtU6-26-sgRNAa- AtU6-26-sgRNAb-  AtU6-26-sgRNAc- SK | AtU6-26-sgRNAa- AtU6-26-sgRNAb- SK |
|  | U6-c-R | TGGATCC**CCCGGG**CTCGAGCGGCCGCCAGT |  |  |
| **Gene Editing Test** | atc3h18-ko-F | TTGAAATCTGAACCGTCC | - | - |
|  | atc3h18-ko-R | TTGAAGAGTCCTACCGTATTTT |  |  |

Note：The underlined nucleotides indicate the homology arm sequences, and bold nucleotides represent cleavage sites.


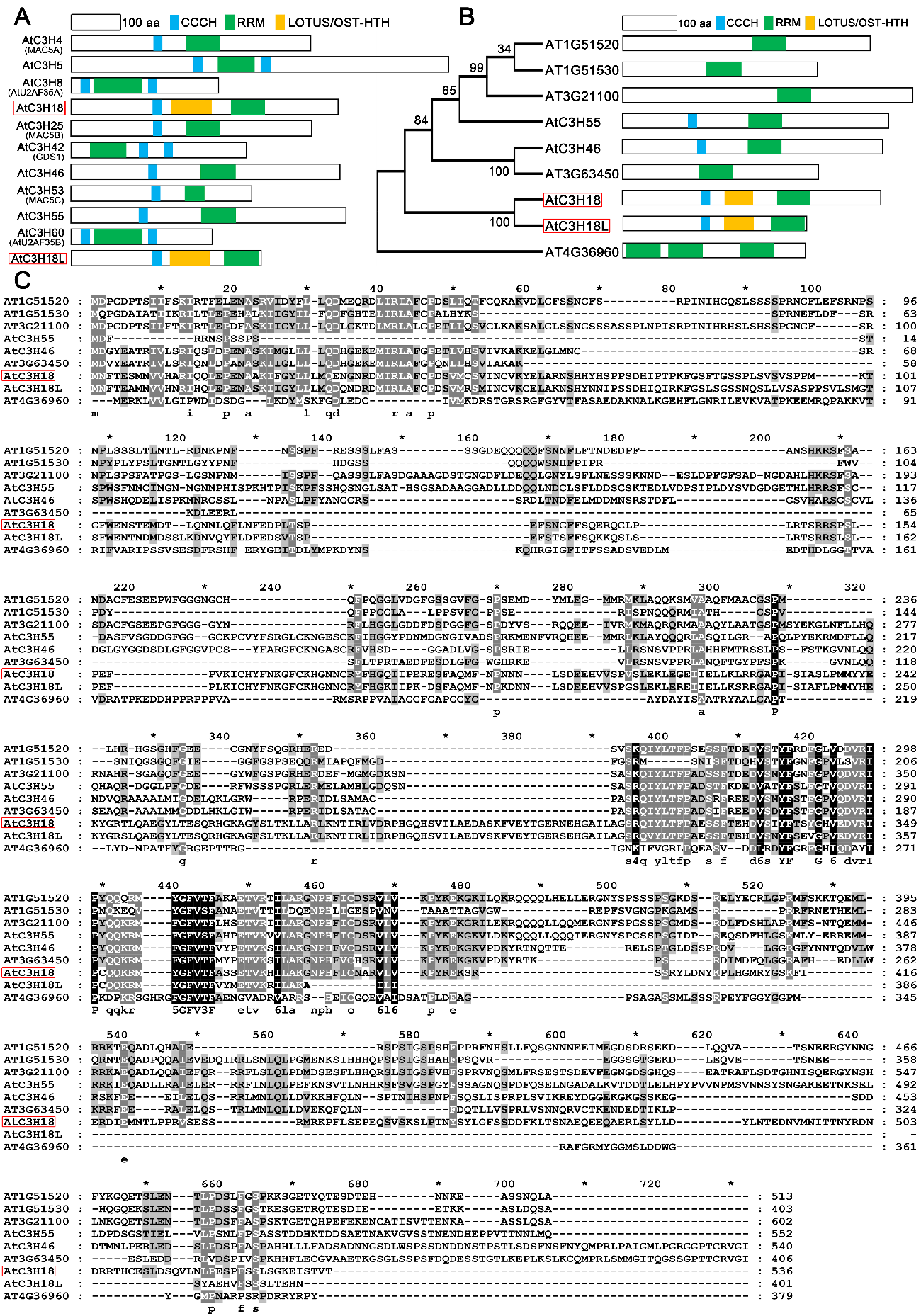


**Supplemental Figure S1.** Phylogenetic and sequence analysis of AtC3H18. A, Protein structures of AtC3H18 and other 10 CCCH zinc finger proteins containing RRM domains. Phylogenetic relationships, protein structures (B) and multiple sequence alignment (C) of AtC3H18 and its putative paralogous in *Arabidopsis*.


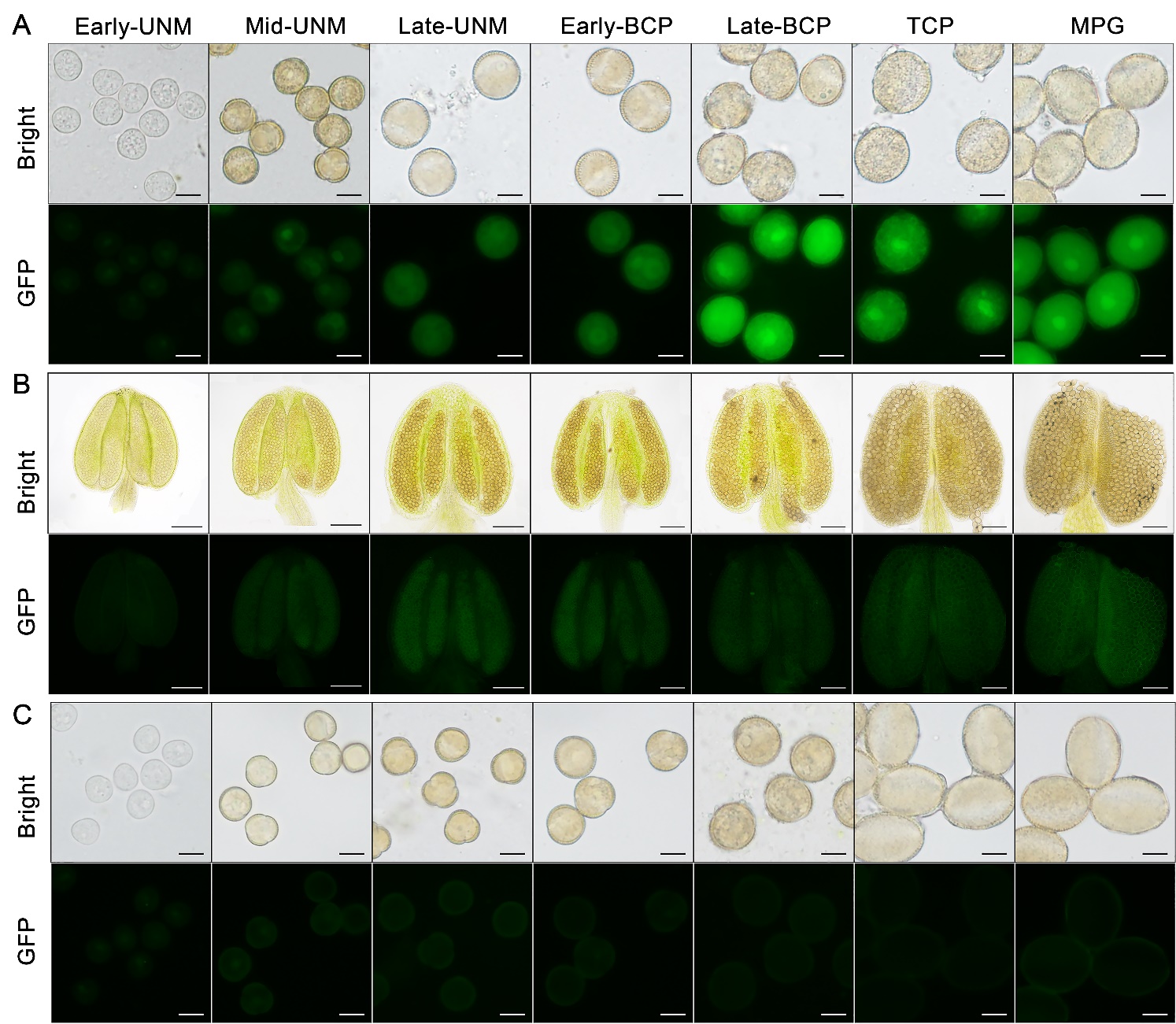


**Supplemental Figure S2.** *AtC3H18* shows the highest expression in late bicellular pollen and tricellular pollen. A, GFP fluorescence intensity of *Pro_AtC3H18_:GFP* transgenic pollen at different developmental stages. The weak spontaneous fluorescence of wild-type anthers (B) and pollen (C). Early/Mid/Late-UNM, early/mid/late uninucleate microspore; Early/Late BCP, early/late bicellular pollen; TCP, tricellular pollen; MPG, mature pollen grain. Bars = 10 μm in (A, C), 100 μm in (B).


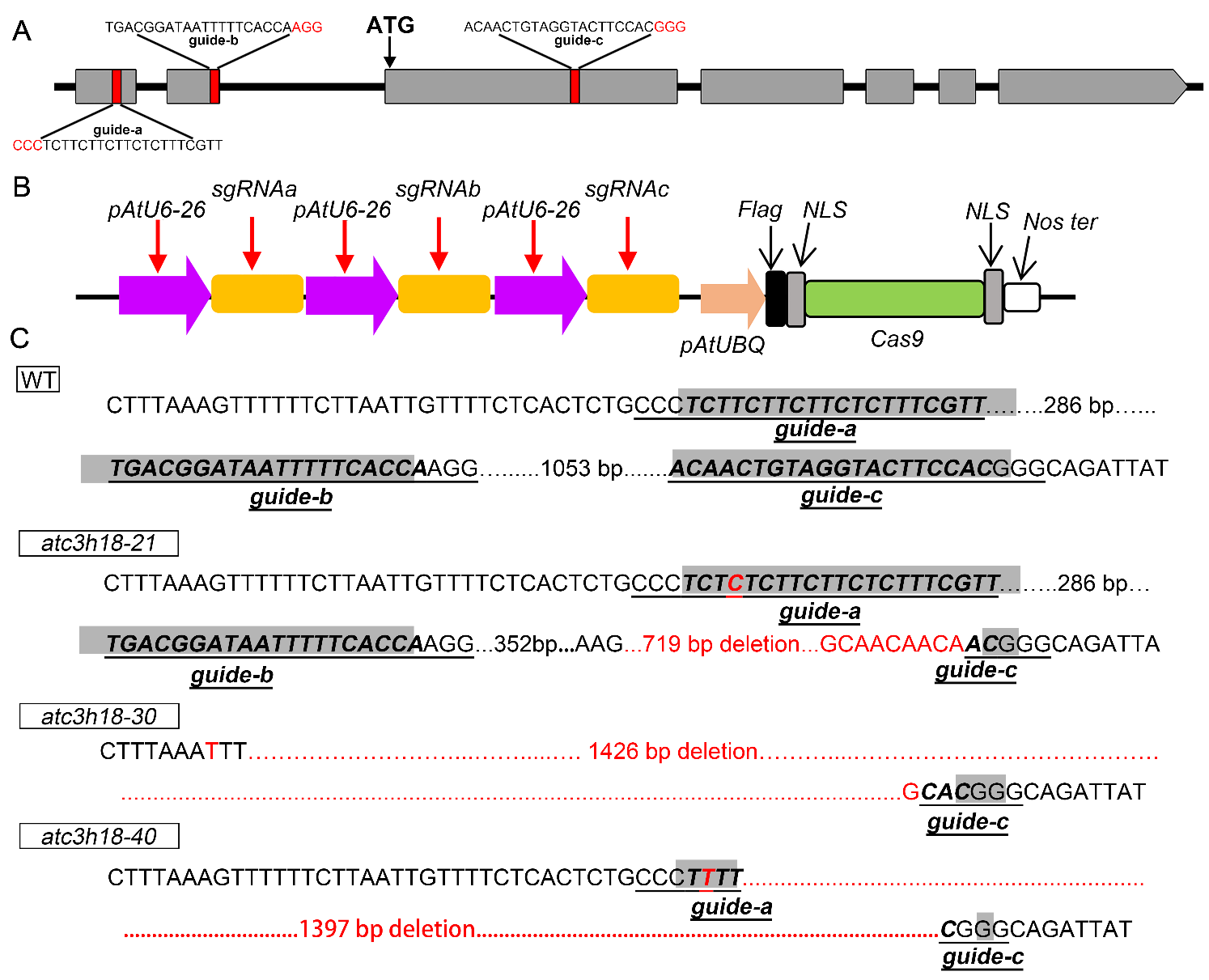


**Supplemental Figure S3.** Generation of the *atc3h18* mutants using CRISPR/Cas9 technology. A, Genomic location of three guide sequences targeting *AtC3H18*. B, Schematic diagram of the CRISPR/Cas9 construct containing a Cas9 expression cassette driven by the promoter of *AtUBQ* and three sgRNA (sgRNAa, sgRNAb and sgRNAc) controlled by the *AtU6-26* promoter. C, Large fragment deletions were detected in *AtC3H18* genomic DNA of homozygous mutants. A 1-bp insertion, a 719-bp deletion and a 9-bp insertion were detected in *AtC3H18* genomic DNA of *atc3h18-21*. A 1-bp substitution, a 1426-bp deletion and a 1-bp insertion were detected in *AtC3H18* genomic DNA of *atc3h18-30*. A 1-bp substitution and a 1397-bp deletion were detected in *AtC3H18* genomic DNA of *atc3h18-40*.


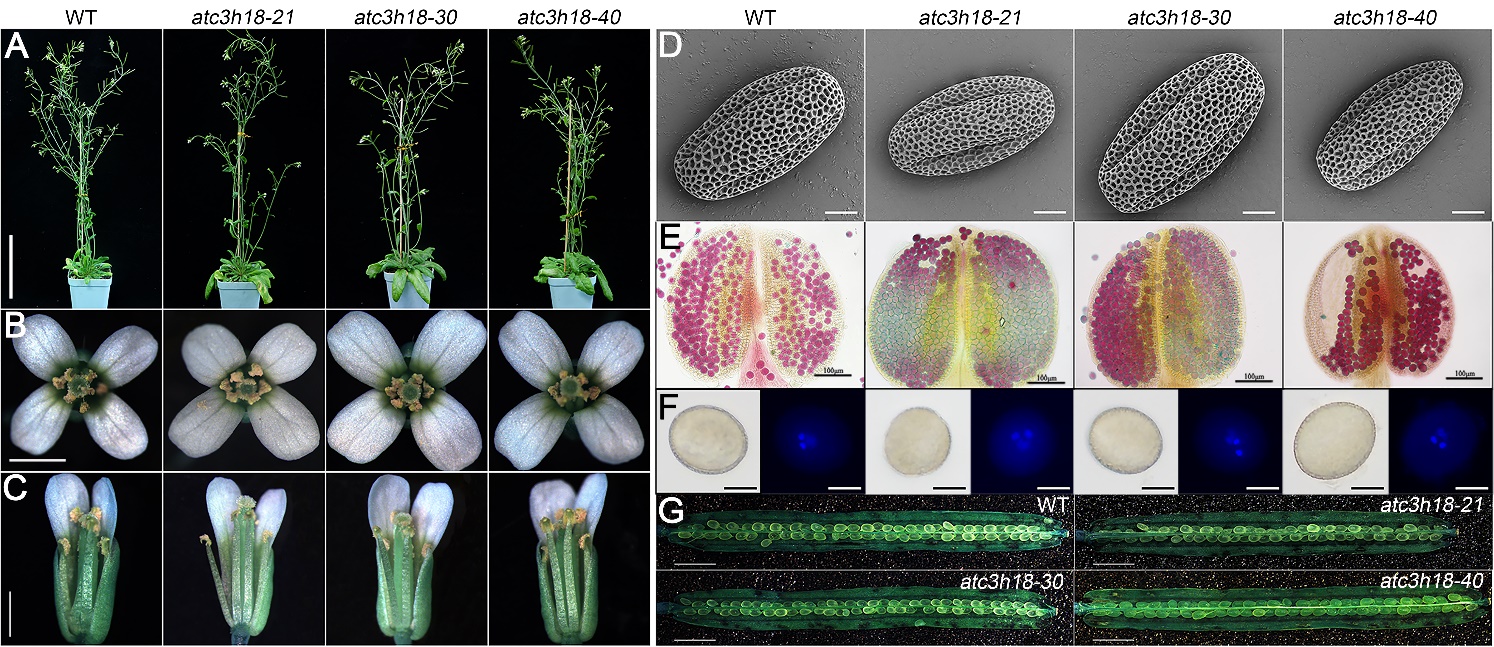


**Supplemental Figure S4.** The *atc3h18* mutants showed no abnormalities during reproductive growth. The plants (A), top (B) and side (C) views of the flowers from wild-type and *atc3h18* mutants. Scanning electron micrographs (D), Alexander (E) and DAPI (F) staining test of mature pollen from wild-type and mutants. Siliques (G) from wild-type and mutants. Bars = 10 cm in (A), 1 mm in (B) and (C), 5 μm in (D), 100 μm in (E), 10 μm in (F) and 2 mm in (G).


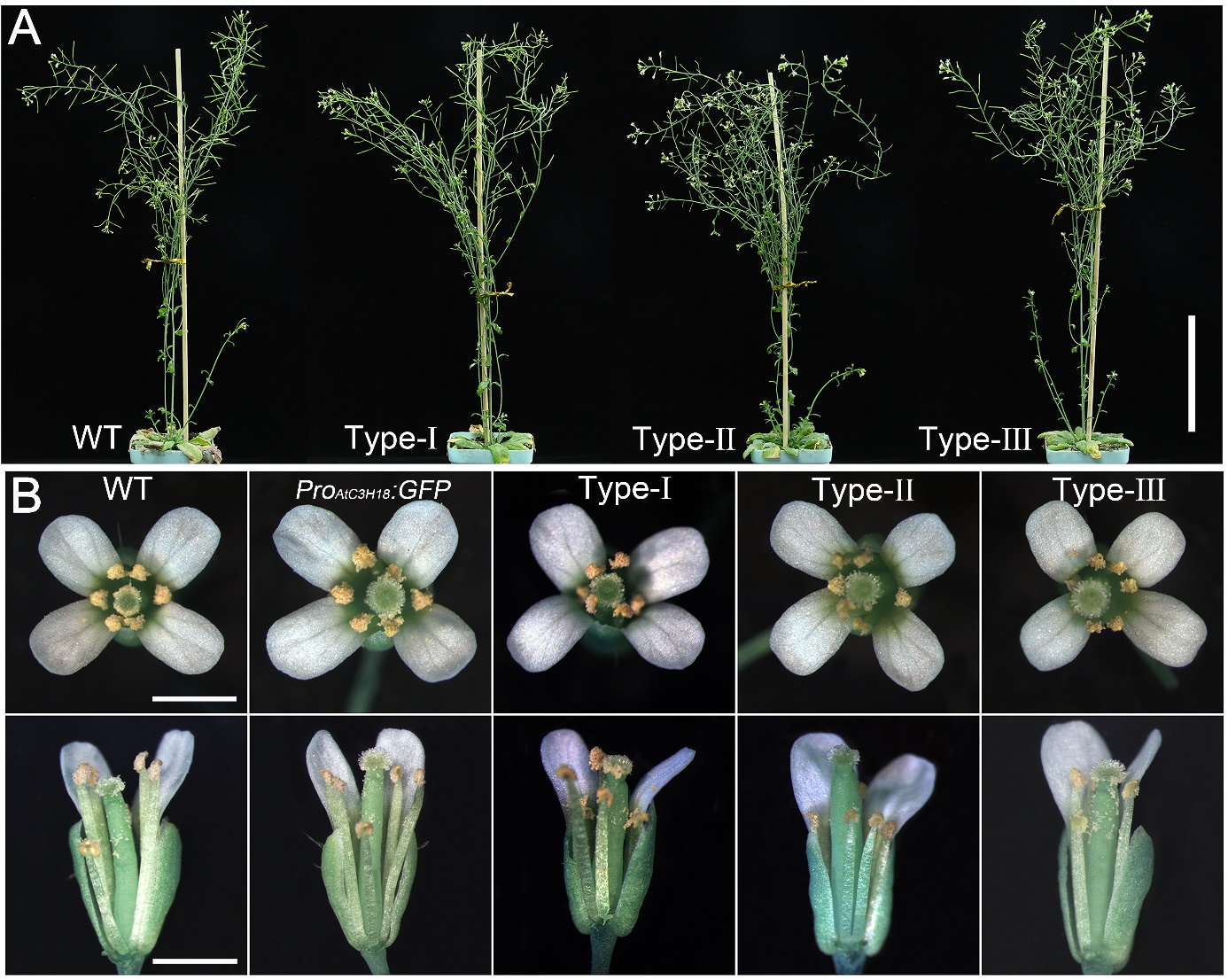


**Supplemental Figure S5.** AtC3H18 overexpression transgenic plants showed normal morphology. A, Representative plant images of wild-type, Type-Ⅰ, Type-Ⅱ, and Type-Ⅲ *Pro_AtC3H18_:AtC3H18-GFP* transgenic plants. Bar = 10 cm. B, Flower top views and side views. Bars = 1 mm.


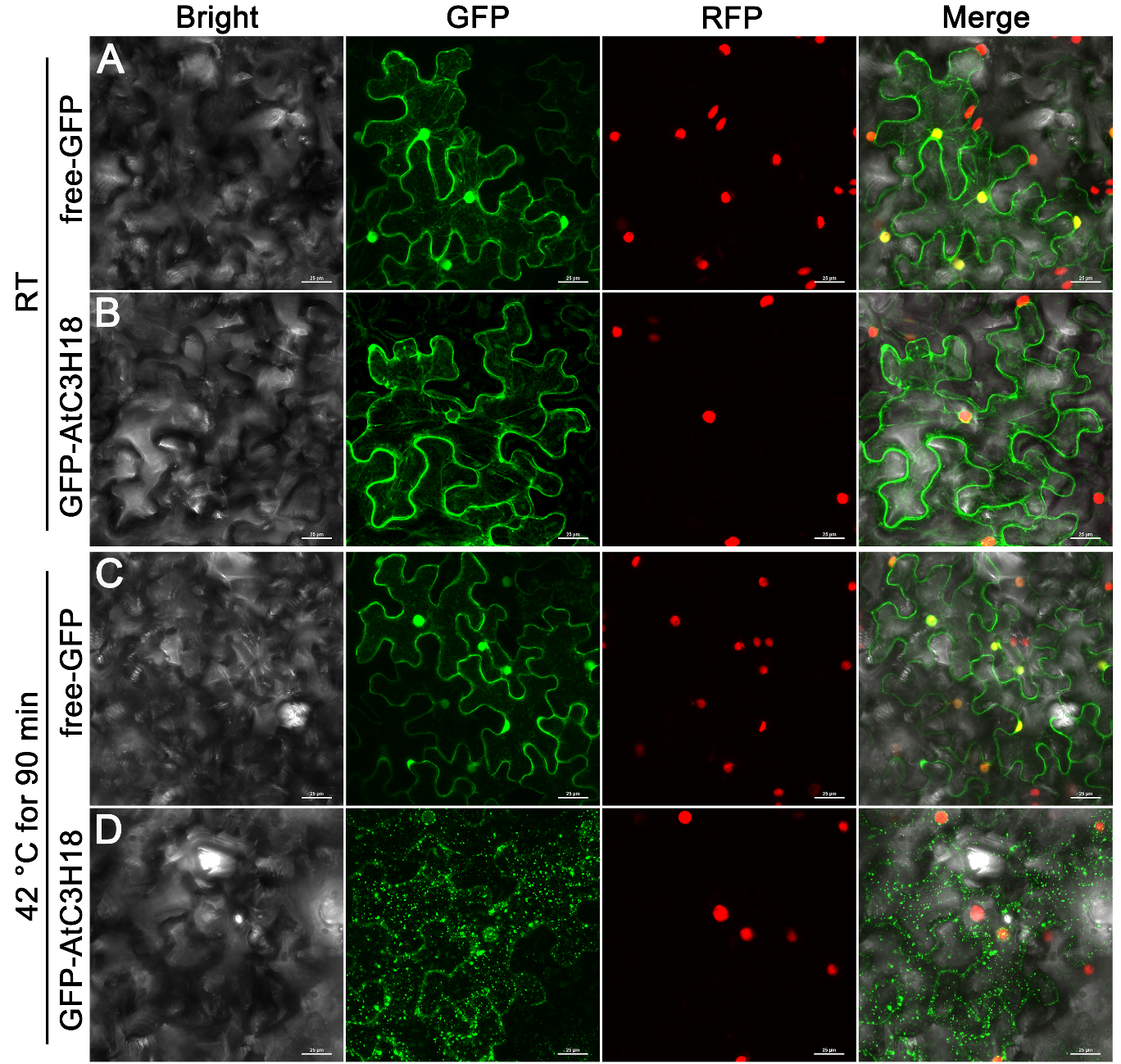


**Supplemental Figure S6.** Heat stress induces re-localization of At3H18 into the cytoplasmic foci. The localization images of free-GFP (A and C) and GFP-AtC3H18 fusion protein (B and D) in *N. benthamiana* leaf epidermal cells at room temperature (RT, A and B) and after heat treatment (C and D), respectively. Pictures represent white field images (Bright), epifluorescence (GFP and RFP) and merged images (Merge). All Images were merged from multiple cutting planes. Bars =25 μm.


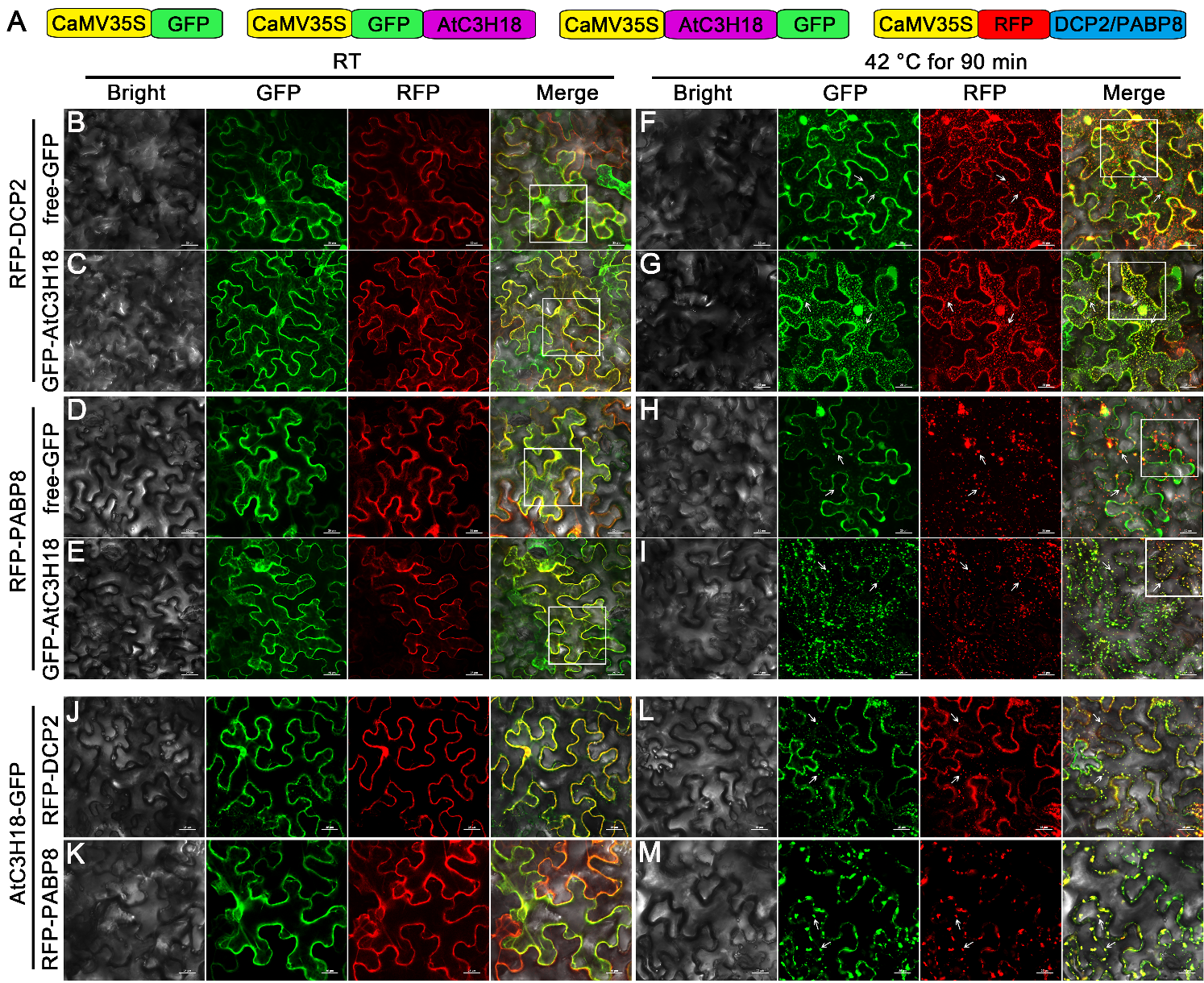


**Supplemental Figure S7.** GFP-AtC3H18 and AtC3H18-GFP fusion proteins can co-localize with PB and SG marker proteins after heat stress. A, Constructs used for the co-localization analyses. Co-localization images of free-GFP and GFP-AtC3H18 fusion protein with RFP-DCP2 (B, C, F and G) or RFP-PABP8 (D, E, H and I) in *N. benthamiana* leaf epidermal cells at room temperature (RT, B**—**E) and after heat stress (F**—**I), respectively. (J**—**M) Co-localization images of AtC3H18-GFP fusion protein with RFP-DCP2 or RFP-PABP8 in *N. benthamiana* leaf epidermal cells at RT and after heat stress. Pictures represent white field images (Bright), epifluorescence (GFP and RFP) and merged images (Merge). The areas depicted by the white frames in (B**—**E and F**—**I) are shown in Fig. 7. All Images were merged from multiple cutting planes. Bars =25 μm.


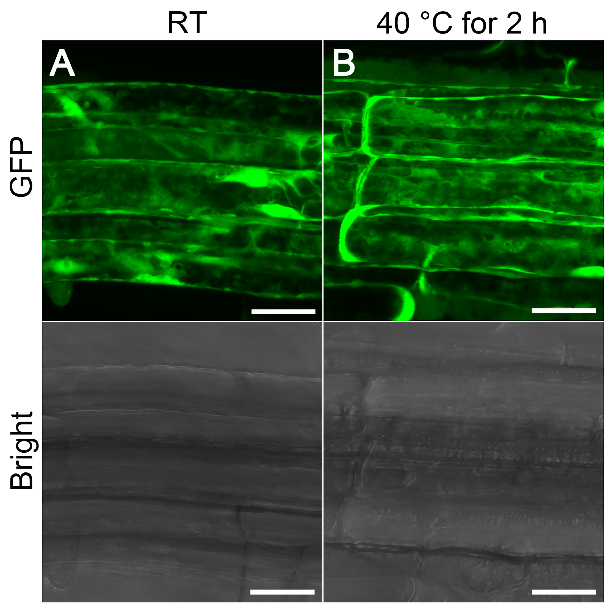


**Supplemental Figure S8.** Cytoplasmic foci cannot be triggered by heat treatment in young roots of *CaMV35S*:*GFP* transgenic seedlings. The localization of free-GFP in *N. benthamiana* leaf epidermal cells at room temperature (RT, A) and after heat stress (B). Pictures represent white field images (Bright) and epifluorescence images (GFP). All Images were merged from multiple cutting planes. Bars = 25 μm.
